# Peripheral retinal arteriolar leakage in giant cell arteritis: a case report

**DOI:** 10.1186/s12348-021-00235-5

**Published:** 2021-01-29

**Authors:** Meleha Ahmad, Andrew R. Carey, Charles G. Eberhart, Sepideh Siadati, Amanda D. Henderson

**Affiliations:** Division of Neuro-Ophthalmology, Wilmer Eye Institute, Johns Hopkins University School of Medicine, 600 N Wolfe Street, Baltimore MD, 21287 USA

**Keywords:** Case report, Cotton wool spot, Giant cell arteritis, Retinal arteriolar leakage, Retinal vascular leakage, Temporal arteritis, Vasculitis

## Abstract

A 76-year old African American female with a history of arteritic ischemic optic neuropathy (AION) secondary to biopsy-proven giant cell arteritis (GCA) presented with unilateral vision loss in her contralateral eye despite high-dose oral steroid treatment. Dilated fundus examination revealed three cotton wool spots. Fluorescein angiography showed slowed arteriolar filling with late staining of small peripheral arteries, consistent with small vessel arteritis. Laboratory tests for alternative vasculitides were negative. Review of her temporal artery biopsy specimen confirmed lymphoplasmacytic inflammation around small adventitial vessels with no destructive granulomatous or leukocytoclastic small vessel vasculitis, consistent with GCA. Our unique case demonstrates peripheral small vessel retinal arteriolar leakage in GCA, which is a rare finding. This association is of interest because GCA is commonly associated with medium to large vessel pathology without small vessel involvement.

## Presentation

**Fig. 1:**
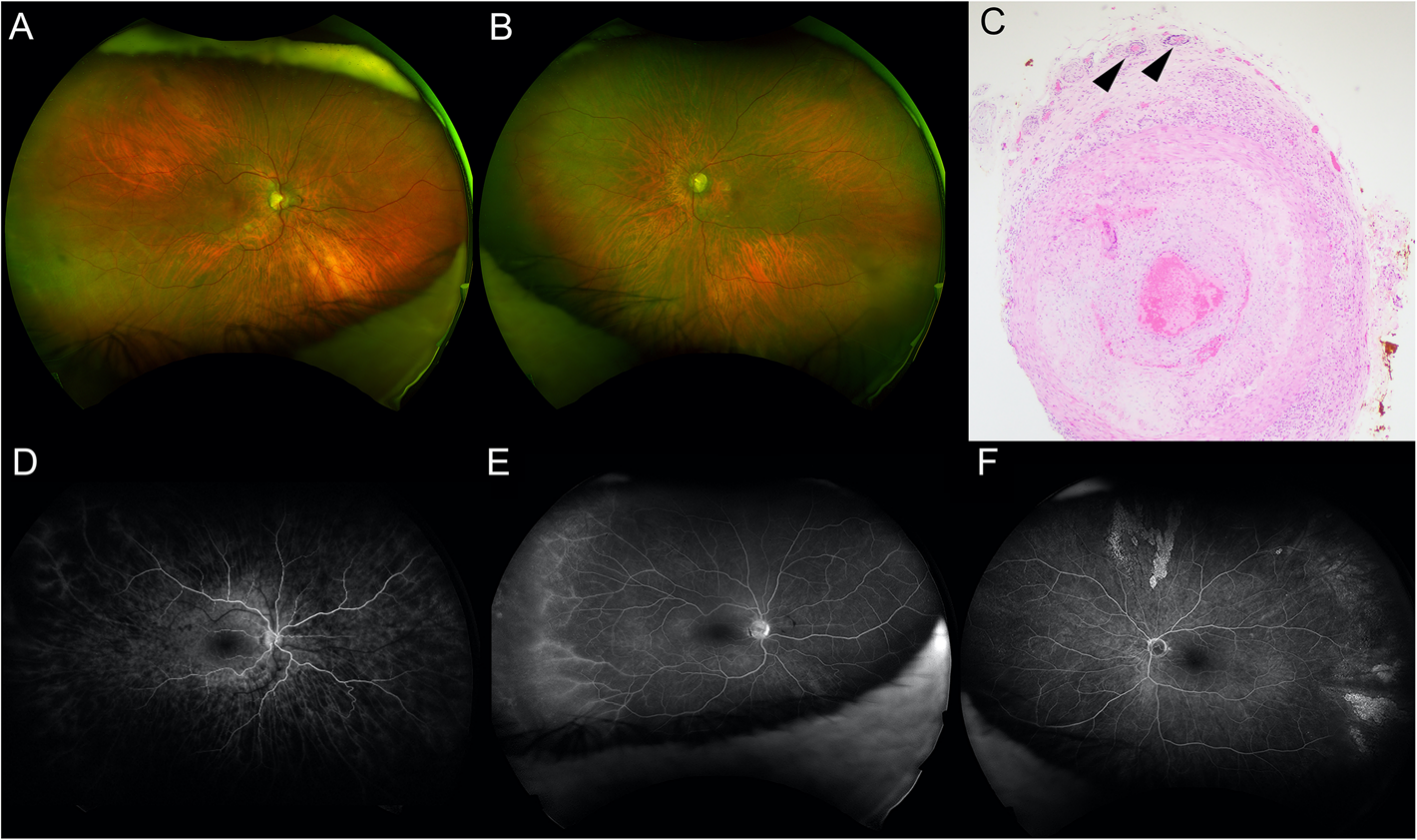
**a** Wide field color fundus photograph of the right eye showing cotton wool spots. **b**: Wide field color fundus photograph of the left eye showing optic nerve pallor and narrowing of the retinal vessels. **d**: Wide field early-frame fluorescein angiography of the right eye showing delayed arteriolar filling and patchy choroidal filling. **e**: Wide field late-frame fluorescein angiography of the right eye showing staining of peripheral small retinal arterioles. **f**: Wide field early-frame fluorescein angiography of the left eye showing patchy choroidal filling defects but no late arteriolar staining. **c**: Temporal artery biopsy demonstrating destructive granulomatous infiltration of the adventitia, media and intima with lymphoplasmacytic inflammation present around small adventitial vessels (arrows)

A 76-year-old woman with a history of hypertension, non-insulin dependent type 2 diabetes, stroke, and arteritic ischemic optic neuropathy (AION) of the left eye due to biopsy-proven giant cell arteritis (GCA) presented with painless, diffuse vision loss in the right eye for the past 10 days despite current high-dose oral steroid treatment (0.75 mg/kg/day prednisone). Diabetes and hypertension were controlled with a recent hemoglobin A1c of 6.4% and blood pressure of 122/74 at recent outpatient visit. Uncorrected visual acuity was decreased from her baseline of 20/40 to 20/70 in the right eye with no improvement with pinhole. Slit lamp examination of the anterior segment was unremarkable except for 2+ nuclear sclerotic cataract and 1+ cortical cataract in the right eye; the left eye was pseudophakic. There was no evidence of inflammation of the anterior or posterior segment of either eye. Dilated fundus examination showed two cotton wool spots in the right eye (Fig. 1a), which had not been noted on recent dilated fundus examinations; there was optic nerve pallor in the left eye in the setting of prior AION (Fig. 1b). Fluorescein angiography demonstrated patchy choroidal non-perfusion and slowed arteriolar filling in the early phase in the right eye (Fig. 1d), likely contributing to her decreased visual acuity, with late peripheral arteriolar leakage in this eye (Fig. 1e) and areas of choroidal non-perfusion in the left eye (Fig. 1f). Inflammatory markers, including erythrocyte sedimentation rate (ESR) and C-reactive protein (CRP), were normal for age. She was treated with high dose intravenous steroids (1 g solumedrol daily × 5 days) followed by 1 mg/kg/day oral prednisone with a slow taper. Workup for other causes of small vessel vasculitis, including anti-neutrophil cytoplasmic antibodies (ANCA), antinuclear antibodies (ANA), lyme and syphilis serologies, was negative. Review of her medications failed to demonstrate any known pharmaceuticals associated with retinal vasculitis or intraocular inflammation. Her temporal artery biopsy specimen demonstrated destructive granulomatous infiltration of the adventitia, media and intima, along with lymphoplasmacytic inflammation around small adventitial vessels without destructive granulomatous or leukocytoclastic small vessel vasculitis, consistent with GCA without evidence of additional vasculitides (Fig. 1c). Steroids were tapered, she was started on tocilizumab by her rheumatologist, and her visual acuity stabilized.

## Discussion

Common retinal ischemic lesions in GCA include central retinal artery occlusion, choroidal ischemic lesions and cotton wool spots (CWS) [1]. These ocular findings are caused primarily by medium vessel vasculitis, except for CWS whose pathogenesis in GCA remains poorly understood. Given the presence of new CWS despite well-controlled diabetes and hypertension, as well as classic findings of GCA in the contralateral eye, the patient’s CWS were felt to be most likely related to GCA rather than other comorbidities such as diabetes and hypertension. Two potential mechanisms for CWS in GCA include local factors, such as elevated fibrinogen levels, contributing to transient retinal microvascular occlusion [2] as well as retrograde or orthograde axoplasmic flow due to upstream occlusion of medium-sized vessels of the posterior ciliary or retinal circulations [3].

Florid retinal vascular leakage and retinal vasculitis are less well described in GCA, with only one prior case reported in the literature (search was conducted using Pubmed database with no language restrictions using keywords: “giant cell arteritis”, “temporal arteritis”, “vasculitis”, “retinal vascular leakage”). Papakostas, et al. described a 75-year old man presenting with subjective blurred vision, with examination demonstrating bilateral CWS, vascular sheathing, and extensive vasculitis with areas of non-perfusion in the more severely affected eye. The patient was diagnosed with GCA based on temporal artery biopsy, with the authors proposing a common pathogenic cause between fundus and temporal artery biopsy findings [4]. Although, strictly speaking, retinal vasculitis requires direct biopsy for definitive diagnosis, these cases of peripheral retinal arteriolar leakage could represent early small-vessel retinal vasculitis in GCA.

Outside of the eye, small vessel vasculitis in GCA has been visited in the pathology literature, with several groups identifying arterial vasculitis in extramural small vessels adjacent to the main temporal artery [5]. This finding may be seen in up to 70% of temporal artery biopsies and may signal a subtype of GCA with disparate clinical features [6]. The mechanism for small vessel involvement in these cases may be distinct from the mechanism occurring in vasculitis affecting distant sites such as the retina, such as a humoral or immunologic cause rather than direct spread  or primary inflammatory cause. The recognition of a potential association between small vessel vasculitis and GCA could add further understanding to the pathogenesis of GCA, which is typically regarded as a large and medium vessel disease.

## Data Availability

Not applicable.

## Abbreviations

AION: Arteritic ischemic optic neuropathy
ANA: Antinuclear antibodies
ANCA: Anti-neutrophil cytoplasmic antibodies
CRP: C-reactive protein
CWS: Cotton wool spot
ESR: Erythrocyte sedimentation rate
GCA: Giant cell arteritis
